# Morphological changes in the arterial pressure waveform following hemodynamic therapies in critical care: A clinical proof‐of‐concept study in older adults

**DOI:** 10.14814/phy2.71032

**Published:** 2026-07-28

**Authors:** F. M. de Raat, M. P. Mulder, A. J. R. De Bie Dekker, A. R. Bouwman, L. J. Montenij, D. W. Donker

**Affiliations:** ^1^ Department of Electrical Engineering Technical University of Eindhoven Eindhoven the Netherlands; ^2^ Department of Anesthesiology Catharina Hospital Eindhoven the Netherlands; ^3^ Cardiovascular and Respiratory Physiology TechMed Centre, University of Twente Enschede the Netherlands; ^4^ Department of Intensive Care Catharina Hospital Eindhoven the Netherlands; ^5^ Department of Anesthesiology Anna Hospital Geldrop the Netherlands; ^6^ Intensive Care Center University Medical Center Utrecht Utrecht the Netherlands

**Keywords:** afterload, arterial waveform analysis, cardiac surgery, contractility, hemodynamic instability, hemodynamic management, intensive care unit, perioperative care, preload

## Abstract

Clinical management of hemodynamic instability remains challenging as underlying mechanisms are complex. A recent in‐silico study showed that peripheral arterial waveform analysis can differentiate deficits in preload, afterload, and contractility. This study explores the clinical applicability of these findings. In this prospective observational study, continuous peripheral arterial blood pressure recordings were obtained from patients during and after cardiac surgery. Retrospectively, we identified isolated therapeutic events with expected physiological effects on preload (fluid bolus), afterload (norepinephrine), or contractility (dobutamine). Waveform morphology was characterized by 23 features. 132 patients were included; 92 fluid boluses, 91 norepinephrine, and 35 dobutamine administrations. Four waveform features differed significantly between the three types of events: augmented pressure (systolic peak minus anacrotic notch, *p* = 0.001), diastolic/systolic area ratio (*p* < 0.001), relative systolic downstroke time (*p* = 0.002), and relative diastolic time (*p* = 0.004). Fluid boluses increased the systolic component, norepinephrine increased the diastolic component, and dobutamine decreased augmented pressure by −3.0 (95% CI –4.5 to −1.6) mmHg (*p* < 0.001). Distinct morphological alterations of the peripheral arterial waveform were observed following a fluid bolus, norepinephrine, and dobutamine, providing a basis for future studies.

## Graphical abstract

In 132 cardiac surgery patients, fluid bolus, norepinephrine, and dobutamine each produced a distinct, recognizable pattern in arterial waveform morphology (systolic area/downstroke time, diastolic area/time, augmented pressure), suggesting arterial waveform analysis is a promising tool for bedside hemodynamic decision support.

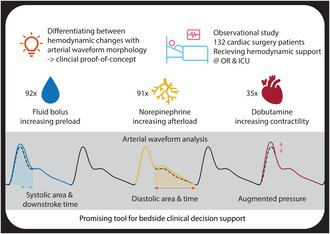

## 1 BACKGROUND

Hemodynamic instability affects approximately 50% of critically ill patients and is strongly associated with multiorgan dysfunction, prolonged intensive care unit (ICU) stay, and increased morbidity and mortality (Abebe et al., 2022; Haase‐Fielitz et al., 2017; Pearse et al., 2012). Effective management is therefore essential to improve patient outcomes (Pinsky et al., 2022; Scott & the APSF Hemodynamic Instability Writing Group, 2024). A key prerequisite for targeted therapy is identifying the underlying hemodynamic mechanism, which can generally be categorized into deficits in preload, afterload, contractility, or a combination (Teboul et al., 2016).

Currently, bedside hemodynamic management often relies on pragmatic, experience‐based approaches reflecting the limited availability of detailed pathophysiological insight at the individual patient level (Malbrain et al., 2016). This highlights the need for a continuous, bedside monitoring tool capable of differentiating between these mechanisms.

We recently demonstrated in an in‐silico study that detailed arterial blood pressure (ABP) waveform analysis can identify deficits in preload, afterload, and contractility (Mulder et al., 2022). Although the physiological basis of arterial waveform morphology has been recognized for decades, clinical studies in critical care have largely focused on single features, most notably the maximum pressure increase during systolic upstroke (dP/dt max), in contexts such as septic shock and hypovolemia (Monge Garcia et al., 2018; Morimont et al., 2012; Sharman et al., 2007; van der Ster et al., 2018). Comprehensive, automated analyses of the full ABP waveform to distinguish between causes of hemodynamic instability are currently lacking.

The aim of this study was to evaluate whether ABP waveform‐derived features can capture changes in distinct hemodynamic mechanisms. To explore this potential, we examined waveform changes associated with three commonly used therapies for hemodynamic support: fluid bolus, norepinephrine, and dobutamine, each expected to predominantly affect preload, afterload, or contractility, respectively. These events served as physiological models to assess the hemodynamic discriminatory ability of ABP waveform features.

## 2 METHODS

### 2.1 Study design and population

This study is a post‐hoc analysis of a subset of observational data from the Hemodynamic Stability Index (HSI) study, conducted between January and November 2023 in the operating room (OR) and intensive care unit (ICU) of the Catharina Teaching Hospital, Eindhoven, The Netherlands. The study was approved by the Medical Ethical Committee of Utrecht (MECU; W2021.072), and informed consent was waived under the General Data Protection Regulation (GDPR).

For the current analysis, patients were eligible if they were ≥18 years old, underwent elective cardiac surgery with postoperative ICU care, received hemodynamic support at the physician's discretion during their OR or ICU stay as a fluid bolus, norepinephrine, and/or dobutamine, and had continuous ABP data available.

### 2.2 Event selection

The study period extended from the start of cardiac surgery until ICU discharge, excluding time on cardiopulmonary bypass. Because deliberately reducing preload, afterload, or contractility is impractical in critically ill patients, we focused on therapies administered during routine care known to predominantly increase these hemodynamic physiological determinants.

The following therapeutic events were analyzed:

1. Fluid bolus (500 or 1000 mL Ringer's lactate within ≤15 min), considered to increase preload (Messina et al., 2022).
2. Norepinephrine (initiation or increase of 0.2–1 mg/h, Fresenius Kabi Nederland BV, catalogue number 04052682059063), considered to increase afterload (Pastuszko et al., 2024).
3. Dobutamine (initiation or increase of 0.5–20 mg/h, Centrafarm B.V., catalogue number 08714632206526), considered to increase contractility (Ashkar et al., 2024).

All therapies were part of routine intra‐ and postoperative hemodynamic management and administered at the discretion of the treating physician. As many dynamically interfering factors are present in the OR, fluid and noradrenaline events were solely selected from the ICU period; for dobutamine this was not possible, as this drug was only used in the OR. Multiple events per patient were included when present.

### 2.3 Waveform analysis

Patient demographics, surgical and ICU characteristics, administration of vasoactive and inotropic medication, and continuous radial ABP data were extracted from the electronic health record and processed using MATLAB (2021b, MathWorks, Inc., USA). ABP data and medication timestamps were automatically synchronized.

For each event, one‐minute waveform segments (sampling frequency 125 Hz) were defined at two time points:

- Baseline: 1 min before the medication administration.
- Post‐event: 5 min after norepinephrine or dobutamine administration, or 10 min after fluid bolus, based on expected pharmacodynamics (Ashkar et al., 2024; Dubin et al., 2017; Pastuszko et al., 2024; Roger et al., 2019).

Events were excluded if other cardio‐ or vasoactive drugs, ventilator settings, or propofol infusion rates were changed within the same time window.

Within each segment, individual heartbeats were detected using the onset of the systolic upstroke. The arterial blood pressure signals were filtered using a fourth‐order zero‐phase Butterworth low‐pass filter with a cutoff frequency of 16 Hz. Individual heartbeats were detected using a slope‐sum function applied to the filtered signal (Sun et al., n.d.; Asgari et al., 2009; Li et al., 2007; Zong et al., 2004). Signal quality was assessed per beat based on physiological plausibility criteria, including bounds on systolic and diastolic pressure, mean arterial pressure, heart rate, and pulse pressure, and beat‐to‐beat consistency with the preceding accepted beat. Signal quality index (SQI; 0 or 1) was calculated per beat based on noise and physiologic plausibility (Sun et al., n.d.; Asgari et al., 2009; Li et al., 2007; Zong et al., 2004). Segments were excluded if beat detection failed or SQI was 0 in >20% of beats, referred to as low signal quality.

Five fiducial points were identified per heartbeat: systolic peak, anacrotic notch, dicrotic notch, diastolic peak, and diastolic end (Alastruey et al., 2023; Mulder et al., 2022). The systolic peak and diastolic end were identified as the maximum and minimum pressure values within each beat. The anacrotic notch, caused by wave reflections, was classified as A‐type when occurring before the systolic peak, detected as a local extremum in the first derivative of the upstroke, or as C‐type when occurring after the systolic peak, detected as a local extremum in the first derivative of the downstroke. If absent, it was positioned at the systolic peak. The dicrotic notch was identified as a local minimum in the first derivative following the systolic peak; if undetectable, it was assigned to one‐third of the beat duration.

Based on prior in‐silico work (Mulder et al., 2022), a selection of 23 waveform features was calculated per beat and averaged over each segment (see File S1, Table A1). Pressures were determined between fiducial points (Nürnberger et al., 2002), and the waveform was divided into systolic (upstroke and downstroke) and diastolic phases (Esper & Pinsky, 2014). For each phase, average slope, duration (as % of total beat), and relative area under the curve were computed. Relative areas excluded portions below diastolic pressure, and the diastolic‐to‐systolic area ratio was calculated (Buckberg et al., 2025).

### 2.4 Statistical analysis

Given the post‐hoc design, the sample size was limited to the available dataset; no formal calculation was performed. Statistical analyses were conducted using IBM SPSS Statistics (version 28.0.1.0, IBM Corp., Armonk, NY, USA). Continuous data are reported as mean ± standard deviation when normally distributed, based visual assessment of the histogram, or median [interquartile range] when not normally distributed. Categorical data are reported as frequency (percentage). Continuous baseline characteristics are compared between the three events with an unpaired *t*‐test or Man‐Whitney *U* test depending on the distribution and categorical baseline characteristics with a Chi‐square test.

The primary analysis assessed changes in the 23 waveform features across the three event types. First, baseline and post‐event values were compared per event using linear models with generalized estimating equations (GEE) to account for repeated measures within patients. Results are presented as estimated marginal means with 95% confidence intervals (CI). Statistical significance was set at *p* < 0.01 to account for the numerous statistical tests. Features were considered specific if significant for only one type of event. Second, changes in waveform features were compared between the three types of events using GEE models.

## 3 RESULTS

**FIGURE 1:**
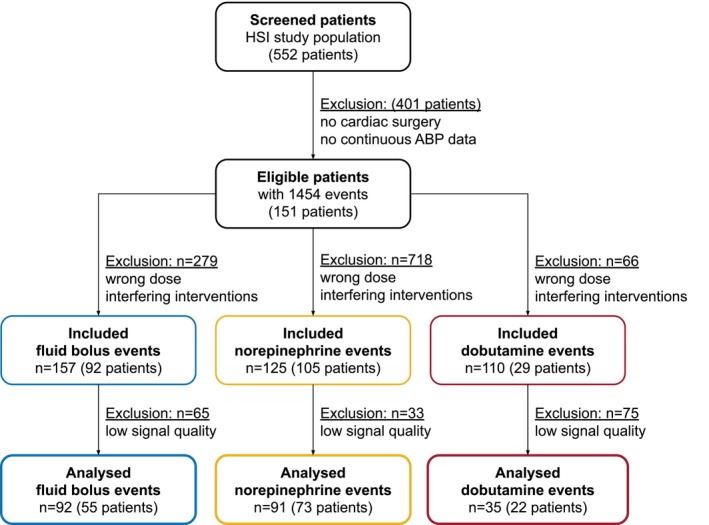
Inclusion and event selection process. ABP, arterial blood pressure; HSI, hemodynamic stability index.

Of the 552 patients enrolled in the HSI study, 151 met eligibility criteria for the current analysis. After excluding 344 fluid bolus, 751 norepinephrine, and 141 dobutamine events due to clinically insignificant dosages, interfering therapies, or low signal quality, 132 unique patients remained. These patients contributed 92 fluid bolus, 91 norepinephrine, and 35 dobutamine events for analysis (Figure 1).

Among fluid bolus events, 54 (59%) occurred in mechanically ventilated patients; all norepinephrine and dobutamine events occurred during mechanical ventilation. Patient demographics and surgical/ICU characteristics are summarized in Table 1.

**TABLE 1:** Characteristics of the study population.

	Event			*p*‐value
	Fluid bolus	Norepinephrine	Dobutamine	
Setting	ICU	ICU	OR	
Patients (*n*)	55	73	22	
Pre‐operative patient characteristics				
Male (%)	88.7	88	84	**0.004**
Age (years)	67 ± 8	66 ± 9	68 ± 8	0.633
BMI (kg/cm^2^)	28.4 [25.5–32.7]	27.8 [24.3–31.5]	26.1 [22.5–27.8]	0.474
APACHE IV	42 ± 12	42 [35–53]	56 [48–72]	**<0.001**
EuroSCORE II	1.3 [1.0–3.0]	1.4 [0.9–2.6]	4.8 ± 2.6	**<0.001**
Creatinine (μmol/L)	89 [78–104]	92 [78–101]	99 [80–113]	0.272
LVEF (%)	55 [50–55]	55 [50–55]	50 [40–50]	0.425
Comorbidities (*n* (%))				
Smoking	12 (21.8)	16 (21.9)	6 (24.0)	**<0.001**
Diabetes	14 (25.9)	14 (19.1)	8 (32.0)	0.302
Hyperlipidaemia	45 (81.8)	58 (79.5)	16 (64.0)	**<0.001**
Hypertension	38 (69.1)	46 (63.0)	10 (40.0)	**<0.001**
COPD	3 (5.5)	5 (6.8)	6 (24.0)	**<0.001**
Previous CVA	5 (9.1)	6 (8.2)	1 (4.0)	**<0.001**
OR characteristics				
Surgery type (*n* (%))				
CABG	38 (69.1)	50 (68.5)	6 (24.0)	0.230
Valve replacement	11 (20)	15 (20.5)	12 (48.0)	0.040
CABG + valve replacement	6 (10.9)	8 (11)	7 (28.0)	0.048
Aortic cross‐clamp time (min)	65 [43–90]	71 ± 33	114 ± 50	0.018
Cardiopulmonary bypass time (min)	98 [75–137]	99 [74–124]	187 ± 56	**<0.001**
ICU characteristics				
ICU stay (days)	0.86 [0.30–1.76]	0.85 [0.27–1.76]	4.20 [2.18–12.12]	**0.002**
Mechanical ventilation (days)	0.16 [0.10–0.31]	0.14 [0.09–0.26]	0.72 [0.18–2.42]	**<0.001**
Complications (*n* (%))	23 (41.8)	29 (39.7)	18 (72.0)	0.048
AF	19 (34.6)	23 (31.5)	12 (48)	0.222
AKI	1 (1.8)	4 (5.5)	8 (32)	**<0.001**
Bleeding	1 (1.8)	2 (2.7)	0 (0.0)	0.783
Cardiac tamponade	2 (3.6)	2 (2.7)	2 (8.0)	0.340
Ischemic complications	3 (5.5)	4 (5.5)	1 (4.0)	0.868

*Note*: Continuous values were statistically compared with an unpaired *t*‐test (normal distribution) or Man‐Whitney *U* test (non‐normal distribution) and categorical values with a Chi‐square test, a *p*‐value <0.01 was considered significant and are depicted in bold.

Abbreviations: AF, atrial fibrillation; AKI, acute kidney injury; APACHE IV, Acute Physiology and Chronic Health Evaluation (version 5); BMI, body mass index; CABG, coronary artery bypass grafting; CVA, cerebrovascular accident; DBP, diastolic blood pressure; EuroSCORE II, European System for Cardiac Operative Risk Evaluation (version 2); ICU, intensive care unit; LVEF, left ventricular ejection fraction; MAP, mean arterial pressure; OR, operating room; SBP, systolic blood pressure.

### 3.1 Waveform changes per event

An overview of all waveform feature changes is provided in File S1, Table A2. A fluid bolus of 500 mL was most commonly administered; only 13 events (14%) involved 1000 mL. Fluid bolus administration predominantly affected the systolic component of the arterial waveform. Significant and specific changes included an increase in relative systolic area of 0.9 (0.5–1.3) mmHg*s (*p* < 0.001), relative systolic downstroke time of 0.8 (0.2–1.3) % (*p* = 0.006), relative anacrotic notch pressure of 3.3 (1.6–4.9) mmHg (p < 0.001), and descending pressure of 2.9 (1.4–4.4) mmHg (*p* < 0.001), and a decrease in heart rate of −1.2 (−1.9–−0.4) beats/min (*p* = 0.002).

Norepinephrine was initiated or increased by a median of 0.3 [0.2–0.4] mg/h and induced significant and specific changes primarily in the diastolic portion of the waveform. The relative diastolic area showed an increase of 0.4 (0.1–0.6) mmHg (*p* < 0.003), the relative diastolic duration an increase of 1.1 (0.4–1.7) % (*p* = 0.002) and the relative dicrotic notch pressure of 1.2 (0.5–2.0) mmHg (*p* = 0.001). The diastolic runoff slope showed a significant and specific decrease of −1.8 (−3.1 to −0.6) mmHg/s (*p* = 0.005).

Dobutamine was increased or initiated with 12.1 ± 4.7 mg/h on average. Its administration resulted solely a significant and specific a decrease in augmented pressure of −3.0 (−4.5 to −1.6) mmHg (*p* < 0.001). Additionally, maximum systolic pressure rise (dP/dt max) increased by 166.2 (48.8–283.6) mmHg/s (*p* = 0.006), although this change was also significant for fluid boluses.

**FIGURE 2:**
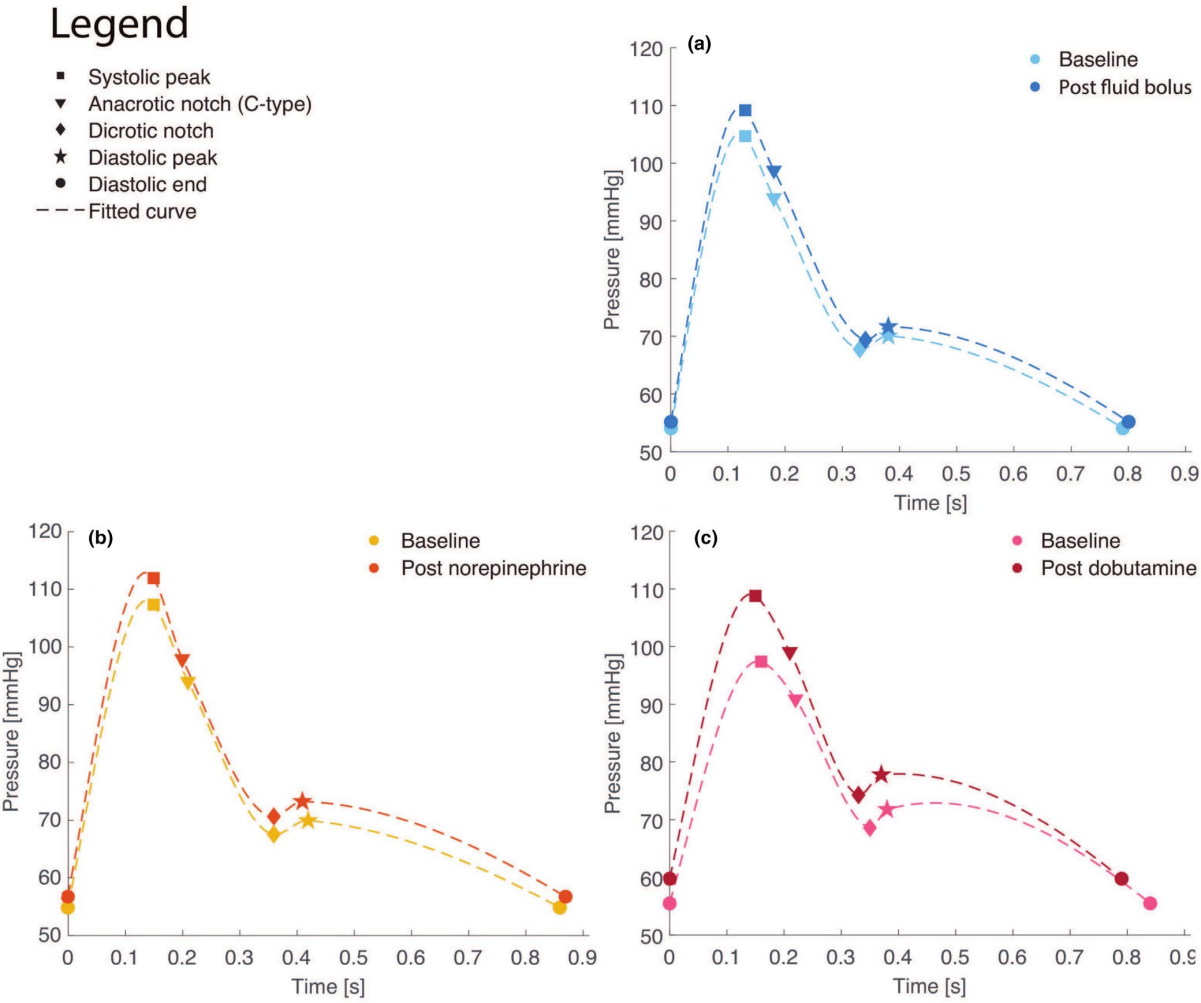
Estimated mean fiducial points for (a) fluid, (b) norepinephrine, and (c) dobutamine events. Estimated marginal mean fiducial points at baseline (light color) and post (dark color) fluid bolus (left), norepinephrine (middle), and dobutamine (right) events. A curve was fitted through the fiducial points for visualization.

Figure 2 illustrates estimated mean fiducial points before and after a fluid bolus (panel A), norepinephrine (panel B), and dobutamine (panel C) with fitted curves for visualization, showing subtle but distinct changes in waveform morphology. Figure A1 displays the same fiducial points relative to diastolic pressure.

### 3.2 Distinctive waveform features

**FIGURE 3:**
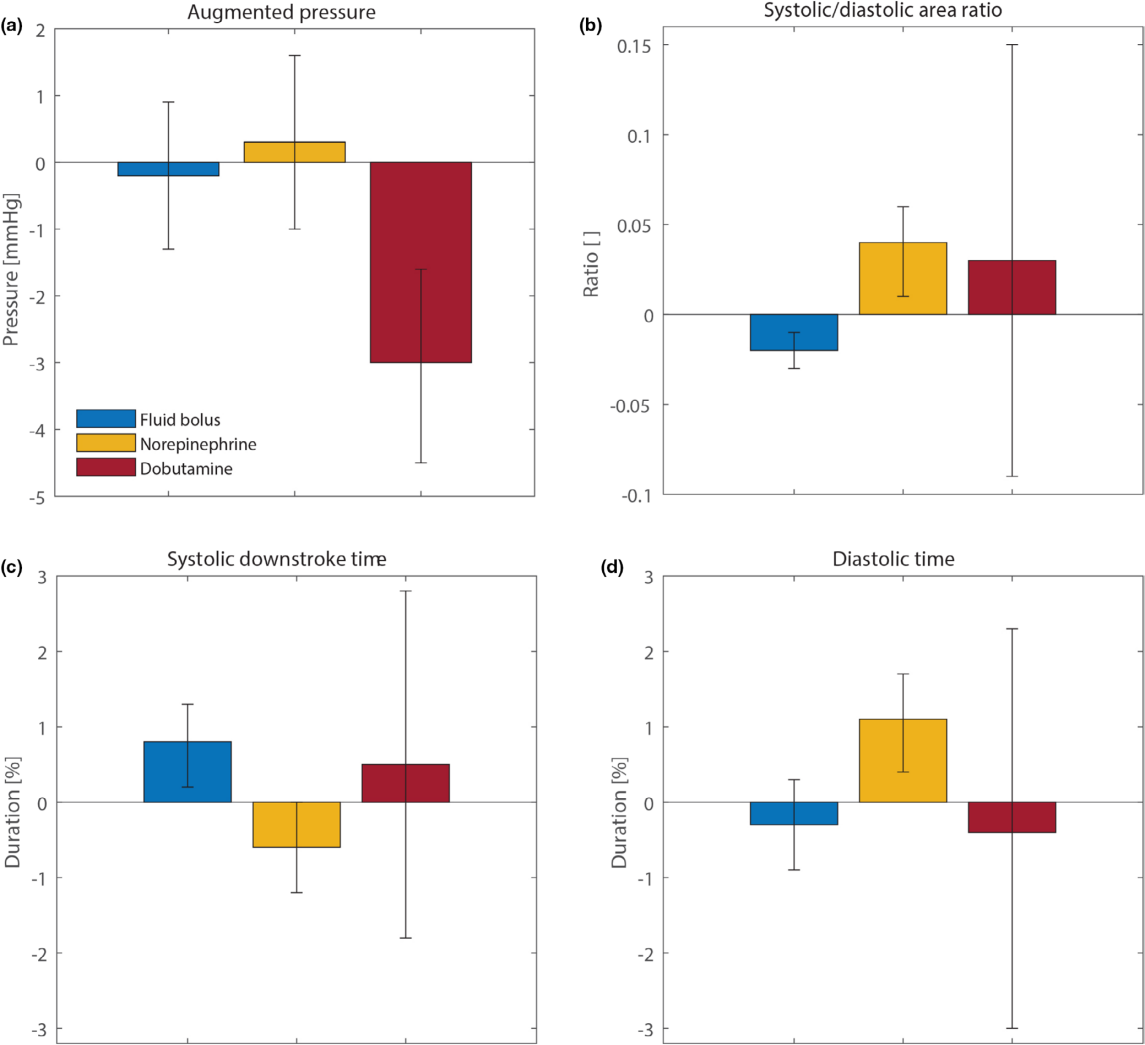
Four distinctive arterial waveform features: (a) augmented pressure (*p* = 0.001), (b) diastolic/systolic area ratio (*p* < 0.001), (c) relative systolic downstroke time (*p* = 0.002) and (d) relative diastolic time (*p* = 0.004). Estimated changes with 95% confidence intervals compared between events; fluid bolus (blue), norepinephrine (yellow) and dobutamine (red).

Importantly, four arterial waveform features were distinctively different between the fluid bolus, norepinephrine, and dobutamine events: augmented pressure (*p* = 0.001), diastolic/systolic area ratio (*p* < 0.001), relative systolic downstroke time (*p* = 0.002), and relative diastolic time (*p* = 0.004) as visualized in Figure 3. A complete overview of all features is provided in File S1, Figures [Link], [Link], and Table A3.

## 4 DISCUSSION

Peripheral ABP waveform features reflected physiological changes following fluid bolus, norepinephrine, and dobutamine administration in critically ill patients during and after cardiac surgery. Four features, augmented pressure, diastolic/systolic area ratio, relative systolic downstroke time, and relative diastolic time, were particularly distinctive across the events. Fluid boluses primarily affected the systolic component, norepinephrine influenced the diastolic component, and dobutamine produced a marked reduction in augmented pressure. These findings confirm that ABP waveform characteristics previously identified in‐silico, including systolic upstroke and downstroke as well as anacrotic and dicrotic notches, can be detected in clinical real‐world data. Notably, they exhibit discriminative potential in hemodynamic events with a known effect on preload, afterload, and contractility.

### 4.1 Distinctive waveform morphology

The systolic and diastolic areas showed specific changes after fluid bolus and norepinephrine, respectively, while remaining unaffected by dobutamine, making the area ratio a key discriminative feature. The systolic area of the ABP pulse is proportional to stroke volume (Jones et al., 1959; Verdouw et al., 1975), which is closely linked to preload via the Frank–Starling mechanism (Delicce & Makaryus, 2023). This principle underlies pulse contour analysis (PCA), which estimates stroke volume from systolic area (Saugel et al., 2021). The overall systolic effect of fluids in this study is consistent with prior in‐silico simulations (Mulder et al., 2022) and clinical studies: fluid challenges in ICU patients increase systolic pressure (Messina et al., 2021), and after mitral valve surgery, improved stroke volume correlates with increased downstroke pressure (Kapadia et al., 2025).

Conversely, norepinephrine increased diastolic area compatible with vasoconstriction, elevating the dicrotic notch and diastolic pressures (Chaudhry et al., 2022). The dicrotic notch, resulting from aortic valve closure and wave reflections, is traditionally associated with arterial stiffness and compliance (Abushouk et al., 2023). In our data, relative dicrotic notch pressure (rDNP) increased significantly after norepinephrine, likely due to higher systemic vascular resistance causing earlier valve closure and enhanced wave reflections (Mulder et al., 2022). These observations align with prior simulations and clinical findings, such as vasoconstrictive drugs in patients with migraine increasing dicrotic notch height relative to pulse pressure (Barzenje et al., 2020; Vaquer et al., 2019).

Changes in relative phase durations were also distinctive: systolic downstroke time increased after fluid bolus, reflecting prolonged ejection with higher preload and stroke volume, whereas norepinephrine prolonged diastole and shortened systole due to earlier valve closure. Both effects also contributed to the observed area ratio changes. Beyond their discriminative value, relative phase durations carry independent physiological significance. Diastolic time is the primary determinant of coronary perfusion, and its prolongation after norepinephrine may reflect a favorable shift in the perfusion‐to‐ejection ratio (Merkus et al., 1999). Expressing these durations relative to total cycle length renders them largely heart rate‐independent, which is particularly relevant given the considerable heart rate variability in ICU patients.

Augmented pressure, defined as the difference between the systolic peak and the anacrotic notch, reflects the contribution of ventricular contractility to the early systolic pressure rise and emerged as a discriminative feature, decreasing specifically after dobutamine. The reduction observed after dobutamine likely reflects a disproportionate rise in the systolic peak driven by more forceful ejection, consistent with the known inotropic mechanism of dobutamine and prior in silico predictions. Similar findings have been reported during dobutamine stress echocardiography, but were primarily attributed to chronotropic rather than inotropic effects (Sharman et al., 2009).

### 4.2 Additional features

Next to the four major distinctive features, the rDNP and dP/dt max are worth discussing, as they might have clinical relevance. As explained above, the vasoconstriction triggered by norepinephrine administration is compatible with a specific increase in rDNP. Nevertheless, rDNP did not show discriminatory abilities between preload, afterload, and contractility in the current study, but may likely still serve as a marker of vascular tone. For example, anesthesia induction reduces afterload and decreases rDNP as reflected in photoplethysmography signals (Coutrot et al., 2019).

The systolic upstroke, quantified as dP/dt max, is a well‐established surrogate of contractility reflecting the more forceful ejection of blood through a steeper increase in blood pressure (de Hert et al., 2006; Vaquer et al., 2019). In our study, dP/dt max increased significantly after dobutamine, consistent with prior clinical (de Hert et al., 2006; Vaquer et al., 2019) and simulation studies (Mulder et al., 2022). However, it also increased after fluid administration, as reported elsewhere (Monge Garcia et al., 2018; Vaquer et al., 2019), indicating that dP/dt max is influenced by multiple cardiovascular factors, not just by cardiac contractility itself. Notably, the dobutamine‐induced increase was threefold greater than that after fluids, suggesting potential pragmatic utility for assessing cardiac function in daily practice when other hemodynamic factors remain stable.

### 4.3 Clinical implications

The advanced arterial waveform analysis applied in this work overlaps with PCA. Yet, the latter merely estimates cardiac output from systolic area, computes pulse pressure variation (PPV), and at times provides dP/dt max as commercially available in some hemodynamic monitoring systems (Monge Garcia et al., 2018; Ostadal et al., 2019). Unlike PCA, our approach evaluates a broader set of detailed morphological features and is not confined to predicting fluid responsiveness as holds for PPV (Michard et al., 2015). Moreover, our advanced analysis comprises beat‐to‐beat features and does not rely on mechanical ventilation‐induced variability, expanding its applicability to spontaneously breathing or non‐ventilated patients (Hatib et al., 2018; Michard, 2024; Saugel et al., 2020).

Rapid identification of the underlying cause of hemodynamic instability, typically reduced preload, afterload, or contractility, is critical in the ICU. The changes in waveform features after events observed in this study may mirror inverse patterns to those seen during hypovolemia, vasoplegia, or myocardial contractile dysfunction. This hypothesis is substantiated with prior clinical and experimental studies that examined both improvement and reduction of preload, afterload, and contractility, and reported opposing changes in specific waveform features (Barzenje et al., 2020; Monge Garcia et al., 2018; Vaquer et al., 2019). Detecting a specific hemodynamic deficit could potentially guide clinicians in selecting appropriate interventions.

Patient‐specific factors such as vascular age and measurement site also influence waveform morphology (Alastruey et al., 2023), limiting cross‐patient comparisons of absolute values. Instead, tracking intra‐patient changes over time, as in this study, may better reflect evolving cardiovascular status. In this sense, monitoring the dicrotic notch has earlier been proposed as a marker of vasomotor tone and predictor of vasopressor responsiveness (Abushouk et al., 2023), while our study aims to provide a more comprehensive analysis of the arterial waveform.

### 4.4 Limitations

This observational analysis has several limitations. Treatment choices were based on clinical judgment and generally inferred hemodynamic deficits rather than comprehensive assessment of the underlying pathophysiology. Advanced hemodynamic monitoring was not routinely used according to local protocol, precluding the availability of cardiac output and systemic vascular resistance data. Consequently, fluid responsiveness could not be assessed, nor could the hemodynamic response to norepinephrine or dobutamine objectively be verified in terms of changes in cardiac output or systemic vascular resistance. Therefore, we cannot assert that these clinical routine interventions exclusively targeted preload, afterload, or contractility deficits, as such specificity lay beyond the scope of this study design.

The classification of events as preload‐, afterload‐, or contractility‐increasing deployed here is a simplification of clinical reality. Norepinephrine was considered a pure vasopressor and dobutamine a pure inotrope, despite known secondary effects such as norepinephrine's inotropic action at higher doses and dobutamine's vasodilatory properties (Ashkar et al., 2024; Pastuszko et al., 2024). Norepinephrine may also increase preload via venous vasoconstriction and mobilization of venous blood (Adda et al., 2021). Furthermore, the inherently complex physiological interdependence of preload, afterload, and contractility complicates attribution of observed waveform changes to a single mechanism. By design, the present study does not attempt to disentangle these mechanisms at a causal level. Instead, it evaluates whether clinically observable morphological waveform changes align with in silico simulated changes under conditions where one determinant is predominantly affected. This approach is consistent with the exploratory, proof‐of‐concept nature of the work and provides a necessary first step toward future mechanistic validation. Despite these intertwined mechanisms, four distinct waveform feature allowed to differentiate between the therapeutic event types.

Waveforms were recorded at the radial artery, which differ from the central aorta in terms of pulse wave amplification, peripheral wave reflection, and the modifying influence of mechanical ventilation, vascular aging, and vasoactive medications on local arterial compliance. However, radial arterial catheterization represents the clinical standard for continuous invasive blood pressure monitoring in the ICU and our analysis focuses on relative morphological changes within each patient overtime. This within‐patient consistency limits, though does not eliminate, the confounding effect of peripheral amplification and compliance differences on our comparisons. Furthermore, formal wave separation analysis, which would allow decomposition of the waveform into forward and backward propogating components, could not be performed, as simultaneous flow velocity measurements and catheter flush‐test data were not available in this dataset. Future studies incorporating Doppler flow measurements at multiple arterial sites, combined with wave separation analysis, would allow more precise attribution of waveform changes to alterations in wave reflection and arterial compliance. A further limitation concerns the dynamic response characteristics of the arterial catheter‐transducer systems. Overdamping attenuates high‐frequency components, reducing fidelity of the dicrotic and anacrotic notches, while underdamping may introduce resonance artifacts inflating systolic peaks and distorting area‐based measurements. Although low‐quality waveform segments were excluded using a validated signal quality index, formal fast‐flush testing was not available. Future studies should incorporate systematic dynamic response assessment to enable post‐hoc correction.

Respiratory variation in waveform morphology was not explicitly filtered; however, averaging features across all beats within each one‐minute segment, encompassing multiple respiratory cycles, is expected to attenuate the majority of respiratory‐driven beat‐to‐beat variation.

Data limitations, lower signal quality, and frequent interfering interventions in the OR resulted in a relatively small sample size for dobutamine events (*n* = 35). Wide confidence intervals for dobutamine may have reduced statistical significance for both intra‐event dobutamine changes and inter‐event comparisons. Nevertheless, these real‐world monitoring data illustrate the feasibility of advanced arterial waveform analysis in routine clinical practice.

### 4.5 Future directions

Future research should move beyond retrospective validation and include a prospective comparison with the thermodilution gold standard for measuring cardiac output and systemic vascular resistance. Incorporating continuous cardiac output, systemic vascular resistance, and dynamic preload indices will be essential to establish mechanistic specificity. Such multimodal datasets would allow formal decomposition of waveform changes into their underlying determinants and enable validation of whether specific waveform features can reliably distinguish between changes in preload, afterload, and contractility. Building on these findings, key waveform features, such as systolic and diastolic area, systolic downstroke and diastolic time, and augmented pressure, could be tracked longitudinally in larger cohorts and covering various ICU populations. Ultimately, prospective interventional studies with controlled titration of vasoactive and inotropic agents will be required to confirm causality.

Integrating advanced ABP waveform analysis into ICU monitoring systems could provide continuous, minimally invasive physiological insight. However, improving algorithm robustness to noise and enhancing signal quality through clinician guidance (Saugel et al., 2020) or automated filtering (Michard, 2024), will be essential.

Moreover, this mechanistic framework of arterial waveform features may subsequently serve as informative inputs for machine learning algorithms. Recent studies have demonstrated their potential to predict hypotension (Hatib et al., 2018), identify sepsis (Mollura et al., 2021), detect central hypovolemia (van der Ster et al., 2018) and predict fluid responsiveness (Gupta et al., 2024; Kamaleswaran et al., 2021). However, such “black box” approaches limit pathophysiological insight.

## 5 CONCLUSIONS

Distinct morphological features of the peripheral arterial waveform were observed following fluid bolus, norepinephrine, and dobutamine in critically ill patients during and after cardiac surgery. These therapeutic hemodynamic alterations were associated with consistent and specific changes in arterial waveform morphology, reflecting differential effects on systolic and diastolic components. While further validation is required, these findings suggest that detailed ABP waveform analysis may provide additional physiological insight and support individualized, mechanism‐based hemodynamic management at the bedside.

## AUTHOR CONTRIBUTIONS

**F. M. de Raat:** Conceptualization; data curation; formal analysis; investigation; methodology; project administration; software; validation. **M. P. Mulder:** Conceptualization; formal analysis; investigation; methodology; validation; visualization. **A. J. R. De Bie Dekker:** Conceptualization; investigation; methodology; supervision. **A. R. Bouwman:** Conceptualization; formal analysis; investigation; supervision. **L. J. Montenij:** Conceptualization; formal analysis; investigation; methodology; supervision; validation. **D. W. Donker:** Conceptualization; formal analysis; investigation; methodology; supervision.

## FUNDING INFORMATION

The authors declare that no funds, grants, or other support were received during the preparation of this manuscript.

## CONFLICT OF INTEREST STATEMENT

The authors F.M.R., A.J.R.B.D., L.J.M., and A.R.B. have declared to have no competing interest. M.P.M. and D.W.D. provide institutional research consultancy to Maquet Critical Care A.R.B. None of these authors receive personal fees.

## ETHICS STATEMENT

This study was in line with the principles of the Declaration of Helsinki. Approval was granted by the institutional review board of the MECU, the Netherlands, on the 8th of April 2021 (W21.094) and was in accordance with the Medical Research Involving Human Subjects Act.

## CONSENT

The need for written informed consent was waived by the local ethical committee and the MECU based on the GDPR. Informed consent for the study was waived by the local ethical committee.

## Supporting information


**File S1:** Figure A1: Relative estimated mean fiducial points. Estimated mean fiducial points before (light color) and after (dark color) fluid bolus (left column, *n* = 92), norepinephrine (middle column, *n* = 91) and dobutamine (right column, *n* = 35) events relative to the diastolic pressure.
Figure A2: Estimate changes in pressures after events.
Figure A3: Estimate changes and 95% confidence intervals in relative pressure after events.

**File S2:** Figure A4: Estimate changes in durations after events.
Figure A5: Estimate changes in areas after events.
Figure A6: Estimate changes in slopes after events.

**File S3:** Table A1: Arterial waveform features definitions.
Table A2: Change in waveform features per event.
Table A3: Change in waveform features compared between events.

## Data Availability

The datasets used and analyzed during the current study are available from the corresponding author on reasonable request.

## References

[phy271032-bib-0001] Abebe, M. M. , Arefayne, N. R. , Temesgen, M. M. , & Admass, B. A. (2022). Incidence and predictive factors associated with hemodynamic instability among adult surgical patients in the post‐anesthesia care unit, 2021: A prospective follow up study. Annals of Medicine and Surgery, 74, 103321. 10.1016/J.AMSU.2022.103321 35145680 PMC8818524

[phy271032-bib-0002] Abushouk, A. , Kansara, T. , Abdelfattah, O. , Badwan, O. , Hariri, E. , Chaudhury, P. , & Kapadia, S. R. (2023). The Dicrotic notch: Mechanisms, characteristics, and clinical correlations. Current Cardiology Reports, 25(8), 807–816. 10.1007/S11886-023-01901-X 37493873

[phy271032-bib-0003] Adda, I. , Lai, C. , Teboul, J. L. , Guerin, L. , Gavelli, F. , & Monnet, X. (2021). Norepinephrine potentiates the efficacy of volume expansion on mean systemic pressure in septic shock. Critical Care, 25(1), 302. 10.1186/S13054-021-03711-5 34419120 PMC8379760

[phy271032-bib-0004] Alastruey, J. , Charlton, P. H. , Bikia, V. , Paliakaite, B. , Hametner, B. , Bruno, R. M. , Mulder, M. P. , Vennin, S. , Piskin, S. , Khir, A. W. , Guala, A. , Mayer, C. C. , Mynard, J. , Hughes, A. D. , Segers, P. , & Westerhof, B. E. (2023). Arterial pulse wave modeling and analysis for vascular‐age studies: A review from VascAgeNet. American Journal of Physiology. Heart and Circulatory Physiology, 325(1), H1–H29. 10.1152/ajpheart.00705.2022 37000606 PMC7614613

[phy271032-bib-0005] Asgari, S. , Bergsneider, M. , & Hu, X. (2009). A robust approach towards recognizing valid arterial blood pressure pulses. IEEE Transactions on Information Technology in Biomedicine, 14(1), 166. 10.1109/TITB.2009.2034845 19884099 PMC2887660

[phy271032-bib-0006] Ashkar, H. , Adnan, G. , Patel, P. , & Makaryus, A. N. (2024). Dobutamine. In xPharm: The comprehensive pharmacology reference (pp. 1–7). Elsevier. 10.1016/B978-008055232-3.61634-4

[phy271032-bib-0007] Barzenje, A. D. , Gjesdal, K. , Winsvold, B. S. , Småstuen, M. C. , Stovner, L. J. , Gravdahl, G. B. , & Nilsen, K. B. (2020). Clinical and vascular responses to propranolol and candesartan in migraine patients: A randomized controlled clinical trial. Cephalalgia Reports, 3. 10.1177/2515816320946491/ASSET/F676E766-53B4-44E0-B4D5-C217BDEAE0FC/ASSETS/IMAGES/LARGE/

[phy271032-bib-0008] Buckberg, G. D. , Fixler, D. E. , Archie, J. P. , & Hoffman, J. I. E. (2025). Experimental subendocardial ischemia in dogs with normal coronary arteries. http://ahajournals.org 10.1161/01.res.30.1.67 5007529

[phy271032-bib-0009] Chaudhry, R. , Miao, J. H. , & Rehman, A. (2022). Physiology, cardiovascular. StatPearls. https://www.ncbi.nlm.nih.gov/books/NBK493197/ 29630249

[phy271032-bib-0010] Coutrot, M. , Joachim, J. , Dépret, F. , Millasseau, S. , Nougué, H. , Matéo, J. , Mebazaa, A. , Gayat, E. , & Vallée, F. (2019). Noninvasive continuous detection of arterial hypotension during induction of anaesthesia using a photoplethysmographic signal: Proof of concept. British Journal of Anaesthesia, 122(5), 605–612. 10.1016/J.BJA.2019.01.037 30916032

[phy271032-bib-0011] de Hert, S. G. , Robert, D. , Cromheecke, S. , Michard, F. , Nijs, J. , & Rodrigus, I. E. (2006). Evaluation of left ventricular function in anesthetized patients using femoral artery dP/dtmax. Journal of Cardiothoracic and Vascular Anesthesia, 20(3), 325–330. 10.1053/J.JVCA.2005.11.006 16750731

[phy271032-bib-0012] Delicce, A. V. , & Makaryus, A. N. (2023). Physiology, frank Starling law. *StatPearls* . https://www.ncbi.nlm.nih.gov/books/NBK470295/ 29262149

[phy271032-bib-0013] Dubin, A. , Lattanzio, B. , & Gatti, L. (2017). The spectrum of cardiovascular effects of dobutamine ‐ from healthy subjects to septic shock patients. Rev. Bras. Ter. Intensiva, 29(4), 490–498. 10.5935/0103-507X.20170068 29340539 PMC5764562

[phy271032-bib-0014] Esper, S. A. , & Pinsky, M. R. (2014). Arterial waveform analysis. Best Practice & Research. Clinical Anaesthesiology, 28(4), 363–380. 10.1016/J.BPA.2014.08.002 25480767

[phy271032-bib-0015] Gupta, C. B. , Basu, D. , Williams, T. K. , Neff, L. P. , Johnson, M. A. , Patel, N. T. , Ganapathy, A. S. , Lane, M. R. , Radaei, F. , Chuah, C. N. , & Adams, J. Y. (2024). Improving the precision of shock resuscitation by predicting fluid responsiveness with machine learning and arterial blood pressure waveform data. Scientific Reports, 14(1), 1–9. 10.1038/s41598-023-50120-5 38278825 PMC10817926

[phy271032-bib-0016] Haase‐Fielitz, A. , Haase, M. , Bellomo, R. , Calzavacca, P. , Spura, A. , Baraki, H. , Kutschka, I. , & Albert, C. (2017). Perioperative hemodynamic instability and fluid overload are associated with increasing acute kidney injury severity and worse outcome after cardiac surgery. Blood Purification, 43(4), 298–308. 10.1159/000455061 28142133

[phy271032-bib-0017] Hatib, F. , Jian, Z. , Buddi, S. , Lee, C. , Settels, J. , Sibert, K. , Rinehart, J. , & Cannesson, M. (2018). Machine‐learning algorithm to predict hypotension based on high‐fidelity arterial pressure waveform analysis. Anesthesiology, 129(4), 663–674. 10.1097/ALN.0000000000002300 29894315

[phy271032-bib-0018] Jones, W. B. , Hefner, L. L. , Bancroft, W. H. , & Klip, W. (1959). Velocity of blood flow and stroke volume obtained from the pressure pulse. Journal of Clinical Investigation, 38, 2087–2090. 10.1172/JCI103986 14407792 PMC441795

[phy271032-bib-0019] Kamaleswaran, R. , Lian, J. , Lin, D. L. , Molakapuri, H. , Nunna, S. , Shah, P. , Dua, S. , & Padman, R. (2021). Predicting volume responsiveness among sepsis patients using clinical data and continuous physiological waveforms. AMIA Annual Symposium Proceedings, 2020, 619. https://pmc.ncbi.nlm.nih.gov/articles/PMC8075451/ 33936436 PMC8075451

[phy271032-bib-0020] Kapadia, V. , Ramu, S. K. , Majeed‐Saidan, M. , Miyasaka, R. , Harb, S. , & Krishnaswamy, A. (2025). Change in Dicrotic notch index predicts outcomes in patients undergoing Transcatheter edge‐to‐edge repair for mitral regurgitation. Structural Heart, 9(2), 100361. 10.1016/j.shj.2024.100361 40124083 PMC11925029

[phy271032-bib-0021] Li, Q. , Mark, R. G. , & Clifford, G. D. (2007). Robust heart rate estimation from multiple asynchronous noisy sources using signal quality indices and a Kalman filter. Physiological Measurement, 29(1), 15–32. 10.1088/0967-3334/29/1/002 18175857 PMC2259026

[phy271032-bib-0022] Malbrain, M. L. N. G. , Huygh, J. , Peeters, Y. , & Bernards, J. (2016). Hemodynamic monitoring in the critically ill: An overview of current cardiac output monitoring methods. F1000Research, 5, F1000 Faculty Rev‐2855. 10.12688/F1000RESEARCH.8991.1 PMC5166586 28003877

[phy271032-bib-0023] Merkus, D. , Kajiya, F. , Vink, H. , Vergroesen, I. , Dankelman, J. , Goto, M. , & Spaan, J. A. E. (1999). Prolonged diastolic time fraction protects myocardial perfusion when coronary blood flow is reduced. Circulation, 100(1), 75–81. 10.1161/01.CIR.100.1.75 10393684

[phy271032-bib-0024] Messina, A. , Bakker, J. , Chew, M. , De Backer, D. , Hamzaoui, O. , Hernandez, G. , Myatra, S. N. , Monnet, X. , Ostermann, M. , Pinsky, M. , Teboul, J. L. , & Cecconi, M. (2022). Pathophysiology of fluid administration in critically ill patients. Intensive Care Medicine Experimental, 10(1), 1–15. 10.1186/S40635-022-00473-4/FIGURES/1 36329266 PMC9633880

[phy271032-bib-0025] Messina, A. , Romano, S. M. , Ozdemirkan, A. , Persona, P. , Tarquini, R. , Cammarota, G. , Romagnoli, S. , Della Corte, F. , Bennett, V. , Monge García, M. I. , Cecconi, M. , & Payen, D. (2021). Multivariable haemodynamic approach to predict the fluid challenge response: A multicentre cohort study. European Journal of Anaesthesiology, 38(1), 22–31. 10.1097/EJA.0000000000001289 32833857

[phy271032-bib-0026] Michard, F. (2024). Towards the automatic detection and correction of abnormal arterial pressure waveforms. Journal of Clinical Monitoring and Computing, 38(4), 749–752. 10.1007/S10877-024-01152-3/FIGURES/1 38573369 PMC11297833

[phy271032-bib-0027] Michard, F. , Chemla, D. , & Teboul, J. L. (2015). Applicability of pulse pressure variation: How many shades of grey? Critical Care, 19(1), 1–3. 10.1186/S13054-015-0869-X/FIGURES/2 25887325 PMC4372274

[phy271032-bib-0028] Mollura, M. , Lehman, L. W. H. , Mark, R. G. , & Barbieri, R. (2021). A novel artificial intelligence based intensive care unit monitoring system: Using physiological waveforms to identify sepsis. Philosophical Transactions of the Royal Society A: Mathematical, Physical and Engineering Sciences, 379(2212), 20200252. 10.1098/RSTA.2020.0252 PMC8805602 34689614

[phy271032-bib-0029] Monge Garcia, M. I. , Jian, Z. , Settels, J. J. , Hunley, C. , Cecconi, M. , Hatib, F. , & Pinsky, M. R. (2018). Performance comparison of ventricular and arterial dP/dt max for assessing left ventricular systolic function during different experimental loading and contractile conditions. Critical Care (London, England), 22(1), 325. 10.1186/s13054-018-2260-1 30486866 PMC6262953

[phy271032-bib-0030] Morimont, P. , Lambermont, B. , Desaive, T. , Janssen, N. , Chase, G. , & D'Orio, V. (2012). Arterial dP/dt max accurately reflects left ventricular contractility during shock when adequate vascular filling is achieved. 10.1186/1471-2261-12-13 PMC3313844 22380679

[phy271032-bib-0031] Mulder, M. P. , Broomé, M. , Donker, D. W. , & Westerhof, B. E. (2022). Distinct morphologies of arterial waveforms reveal preload‐, contractility‐, and afterload‐deficient hemodynamic instability: An in silico simulation study. Physiological Reports, 10(7). 10.14814/PHY2.15242 PMC9004248 35412023

[phy271032-bib-0032] Nürnberger, J. , Keflioglu‐Scheiber, A. , Opazo Saez, A. M. , Wenzel, R. R. , Philipp, T. , & Schäfers, R. F. (2002). Augmentation index is associated with cardiovascular risk. Journal of Hypertension, 20(12), 2407–2414. 10.1097/00004872-200212000-00020 12473865

[phy271032-bib-0033] Ostadal, P. , Vondrakova, D. , Krüger, A. , Janotka, M. , & Naar, J. (2019). Continual measurement of arterial dP/dtmax enables minimally invasive monitoring of left ventricular contractility in patients with acute heart failure. Critical Care, 23(1), 364. 10.1186/S13054-019-2654-8 31752966 PMC6869259

[phy271032-bib-0034] Pastuszko, O. , Bartusik‐Aebisher, D. , & Aebisher, D. (2024). Norepinephrine. In The biochemical guide to hormones (pp. 119–124). Nova Science Publishers. 10.37573/9781585286720.149

[phy271032-bib-0035] Pearse, R. M. , Moreno, R. P. , Bauer, P. , Pelosi, P. , Metnitz, P. , Spies, C. , Vallet, B. , Vincent, J. L. , Hoeft, A. , Rhodes, A. , & European Surgical Outcomes Study (EuSOS) group for the Trials groups of the European Society of Intensive Care Medicine and the European Society of Anaesthesiology . (2012). Mortality after surgery in Europe: A 7 day cohort study. Lancet, 380(9847), 1059–1065. 10.1016/S0140-6736(12)61148-9 22998715 PMC3493988

[phy271032-bib-0036] Pinsky, M. R. , Cecconi, M. , Chew, M. S. , De Backer, D. , Douglas, I. , Edwards, M. , Hamzaoui, O. , Hernandez, G. , Martin, G. , Monnet, X. , Saugel, B. , Scheeren, T. W. L. , Teboul, J. L. , & Vincent, J. L. (2022). Effective hemodynamic monitoring. Critical Care, 26(1), 1–10. 10.1186/S13054-022-04173-Z 36171594 PMC9520790

[phy271032-bib-0037] Roger, C. , Zieleskiewicz, L. , Demattei, C. , Lakhal, K. , Piton, G. , Louart, B. , Constantin, J. M. , Chabanne, R. , Faure, J. S. , Mahjoub, Y. , Desmeulles, I. , Quintard, H. , Lefrant, J. Y. , Muller, L. , & AzuRea Group . (2019). Time course of fluid responsiveness in sepsis: The fluid challenge revisiting (FCREV) study. Critical Care, 23(1), 1–10. 10.1186/S13054-019-2448-Z/FIGURES/4 31097012 PMC6524325

[phy271032-bib-0038] Saugel, B. , Kouz, K. , Meidert, A. S. , Schulte‐Uentrop, L. , & Romagnoli, S. (2020). How to measure blood pressure using an arterial catheter: A systematic 5‐step approach. Critical Care, 24(1), 1–10. 10.1186/S13054-020-02859-W/FIGURES/4 32331527 PMC7183114

[phy271032-bib-0039] Saugel, B. , Kouz, K. , Scheeren, T. W. L. , Greiwe, G. , Hoppe, P. , Romagnoli, S. , & de Backer, D. (2021). Cardiac output estimation using pulse wave analysis—Physiology, algorithms, and technologies: A narrative review. British Journal of Anaesthesia, 126(1), 67–76. 10.1016/j.bja.2020.09.049 33246581

[phy271032-bib-0040] Scott, M. J. , & the APSF Hemodynamic Instability Writing Group . (2024). Perioperative patients with hemodynamic instability: Consensus recommendations of the Anesthesia Patient Safety Foundation. Anesthesia and Analgesia, 138(4), 713–724. 10.1213/ANE.0000000000006789 38153876 PMC10916753

[phy271032-bib-0041] Sharman, J. E. , Davies, J. E. , Jenkins, C. , & Marwick, T. H. (2009). Augmentation index, left ventricular contractility, and wave reflection. Hypertension, 54(5), 1099–1105. 10.1161/HYPERTENSIONAHA.109.133066 19720955

[phy271032-bib-0042] Sharman, J. E. , Qasem, A. M. , Hanekom, L. , Gill, D. S. , Lim, R. , & Marwick, T. H. (2007). Radial pressure waveform dP/dt max is a poor indicator of left ventricular systolic function. European Journal of Clinical Investigation, 37(4), 276–281. 10.1111/J.1365-2362.2007.01784.X 17373963

[phy271032-bib-0043] Sun, J. X. , Reisner, A. T. , & Mark, R. G. (n.d.). A Signal Abnormality Index for Arterial Blood Pressure Waveforms.

[phy271032-bib-0044] Teboul, J. L. , Saugel, B. , Cecconi, M. , de Backer, D. , Hofer, C. K. , Monnet, X. , Perel, A. , Pinsky, M. R. , Reuter, D. A. , Rhodes, A. , Squara, P. , Vincent, J. L. , & Scheeren, T. W. (2016). Less invasive hemodynamic monitoring in critically ill patients. Intensive Care Medicine, 42(9), 1350–1359. 10.1007/S00134-016-4375-7 27155605

[phy271032-bib-0045] van der Ster, B. J. P. , Westerhof, B. E. , Stok, W. J. , & van Lieshout, J. J. (2018). Detecting central hypovolemia in simulated hypovolemic shock by automated feature extraction with principal component analysis. Physiological Reports, 6(22), e13895. 10.14814/PHY2.13895 30488597 PMC6429974

[phy271032-bib-0046] Vaquer, S. , Chemla, D. , Teboul, J. L. , Ahmad, U. , Cipriani, F. , Oliva, J. C. , Ochagavia, A. , Artigas, A. , Baigorri, F. , & Monnet, X. (2019). Influence of changes in ventricular systolic function and loading conditions on pulse contour analysis‐derived femoral dP/dt max. Annals of Intensive Care, 9(1), 1–10. 10.1186/S13613-019-0537-4/FIGURES/2 PMC6542880 31147862

[phy271032-bib-0047] Verdouw, P. D. , Beaune, J. , Roelandt, J. , & Hygenholtz, P. G. (1975). Stroke volume from central aortic pressure? A critical assessment of the various formulae as to their clinical value. Basic Research in Cardiology, 70(4), 377–389. 10.1007/BF01914334 1191206

[phy271032-bib-0048] Zong, W. , Moody, G. B. , & Mark, R. G. (2004). Reduction of false arterial blood pressure alarms using signal quality assessment and relationships between the electrocardiogram and arterial blood pressure. Medical & Biological Engineering & Computing, 42(5), 698–706. 10.1007/BF02347553/METRICS 15503972

