# Supplementary material for: Morphological changes in the arterial pressure waveform following hemodynamic therapies in critical care: A clinical proof‐of‐concept study in older adults
Source: Physiol Rep. 2026 Jul 28;14(15):e71032. doi: 10.14814/phy2.71032 (PMC13415750; doi:10.14814/phy2.71032)

# Additional figures (part 1)

Figure A1 – Relative estimated mean fiducial points


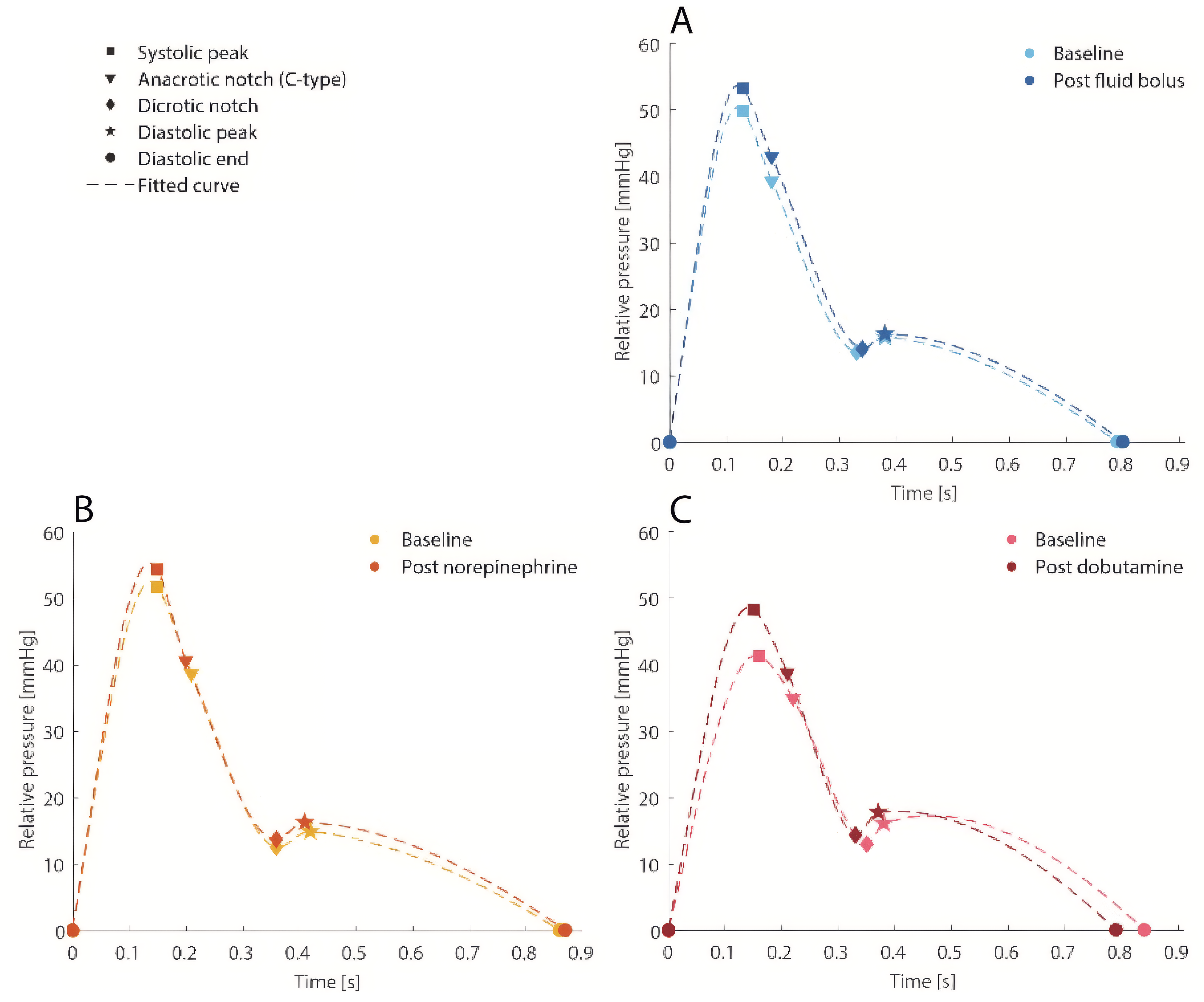


*Estimated mean fiducial points before (light colour) and after (dark colour) fluid bolus (left column, n=92), norepinephrine (middle column, n=91) and dobutamine (right column, n=35) events relative to the diastolic pressure.*

Figure A2 – Estimate changes in pressures after events


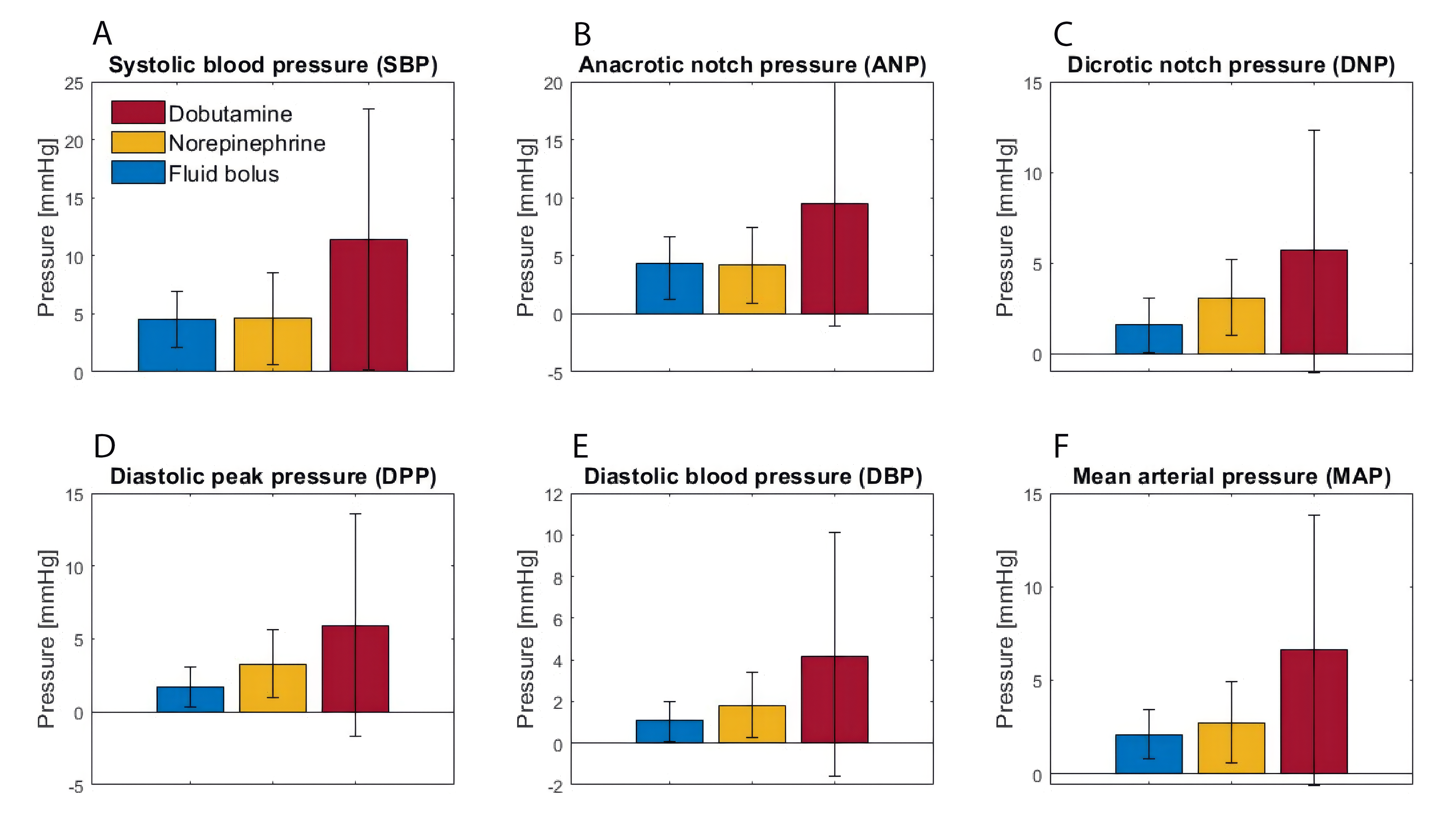


Figure A3 – Estimate changes and 95% confidence intervals in relative pressure after events


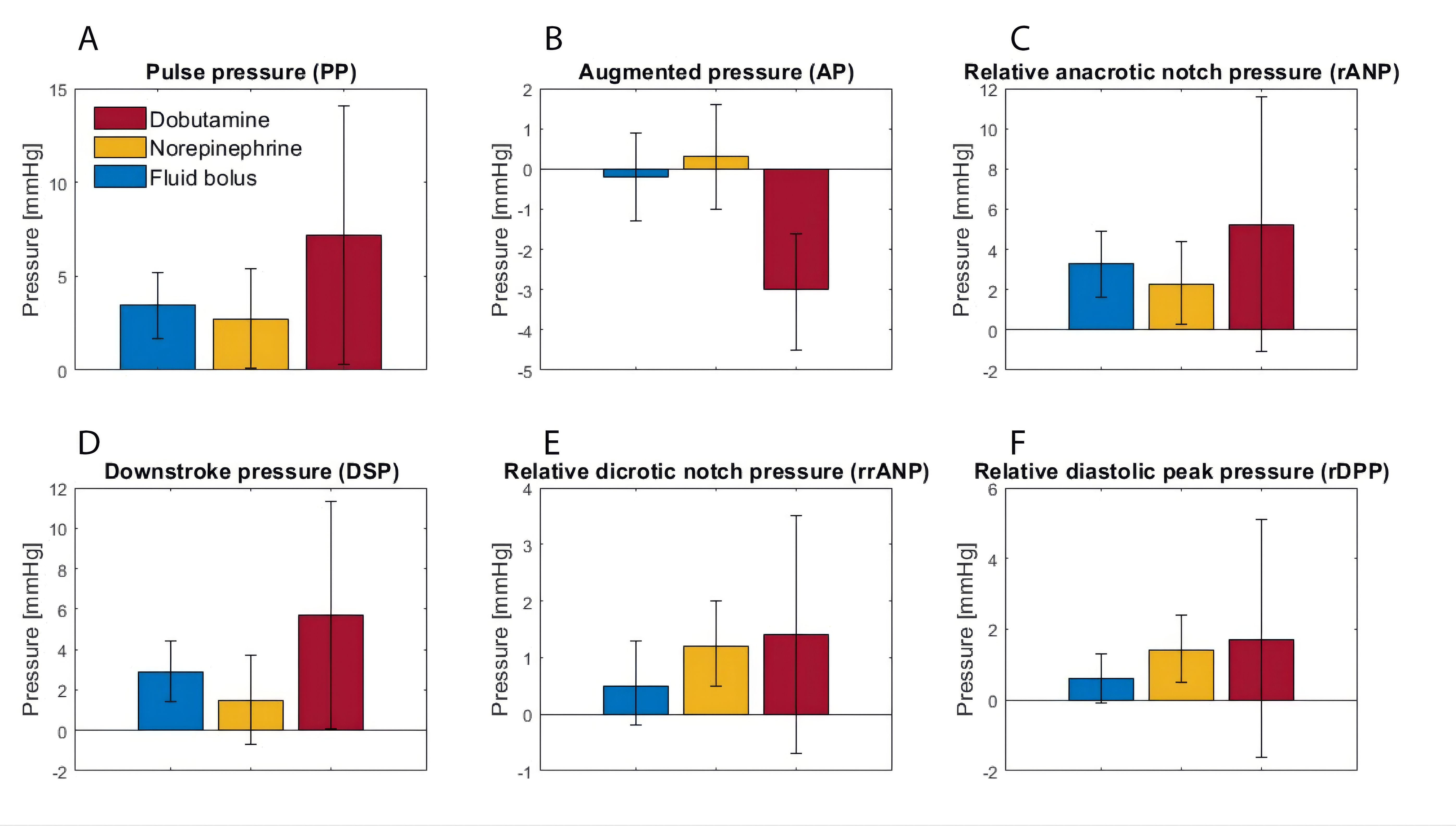

Supplement: Supplementary file 1 — File S1: Figure A1: Relative estimated mean fiducial points. Estimated mean fiducial points before (light color) and after (dark color) fluid bolus (left column, n = 92), norepinephrine (middle column, n = 91) and dobutamine (right column, n = 35) events relative to the diastolic pressure. Figure A2: Estimate changes in pressures after events. Figure A3: Estimate changes and 95% confidence intervals in relative pressure after events. [file PHY2-14-e71032-s003.docx]
