# Supplementary material for: Morphological changes in the arterial pressure waveform following hemodynamic therapies in critical care: A clinical proof‐of‐concept study in older adults
Source: Physiol Rep. 2026 Jul 28;14(15):e71032. doi: 10.14814/phy2.71032 (PMC13415750; doi:10.14814/phy2.71032)

Additional figures (part 2)

Figure A4 – Estimate changes in durations after events


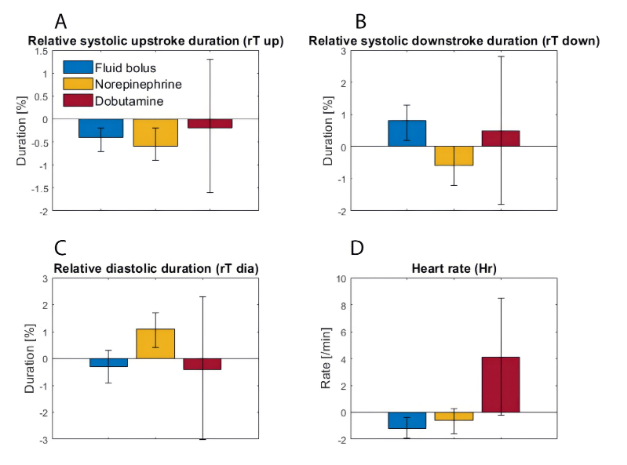


Figure A5 – Estimate changes in areas after events


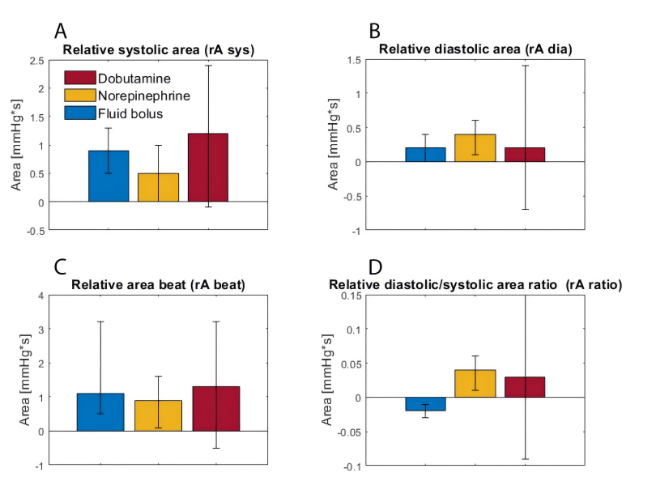


Figure A6 – Estimate changes in slopes after events


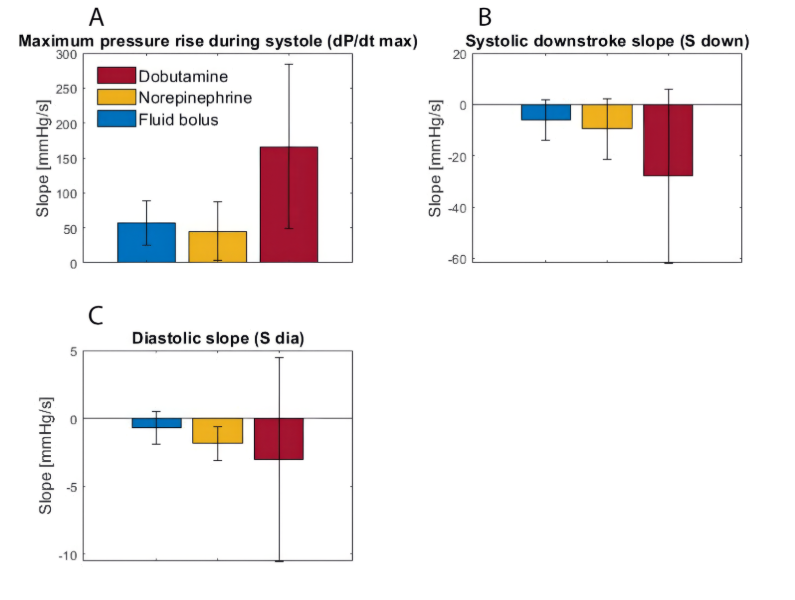

Supplement: Supplementary file 2 — File S2: Figure A4: Estimate changes in durations after events. Figure A5: Estimate changes in areas after events. Figure A6: Estimate changes in slopes after events. [file PHY2-14-e71032-s002.docx]
