# Supplementary material for: Morphological changes in the arterial pressure waveform following hemodynamic therapies in critical care: A clinical proof‐of‐concept study in older adults
Source: Physiol Rep. 2026 Jul 28;14(15):e71032. doi: 10.14814/phy2.71032 (PMC13415750; doi:10.14814/phy2.71032)
Supplement: Supplementary file 3 — File S3: Table A1: Arterial waveform features definitions. Table A2: Change in waveform features per event. Table A3: Change in waveform features compared between events. [file PHY2-14-e71032-s001.docx]

Additional tables

Table A1 – Arterial waveform features definitions

| Feature | Abbreviation | Unit | Definition |
| --- | --- | --- | --- |
| Fiducial points / pressures |  |  |  |
| Systolic blood pressure | SBP | mmHg | max (p)max (p) |
| Anacrotic notch pressure | ANP | mmHg | p(anacrotic notch)p(anacrotic notch) |
| Dicrotic notch pressure | DNP | mmHg | p(dicrotic notch)p(dicrotic notch) |
| Diastolic peak pressure | DPP | mmHg | max (p(dicrotic notch:end))max (p(dicrotic notch:end)) |
| Diastolic blood pressure | DBP | mmHg | min (p)min (p) |
| Mean arterial pressure | MAP | mmHg | mean (p)mean (p) |
| Relative pressures |  |  |  |
| Pulse pressure | PP | mmHg | SBP−DBPSBP−DBP |
| Augmented pressure | AP | mmHg | if A-type anacrotic notch:  SBP−ANPSBP−ANP  if C-type anacrotic notch:  ANP−SBPANP−SBP |
| Relative anacrotic notch pressure | rANP | mmHg | ANP−DBPANP−DBP |
| Downstroke pressure | DSP | mmHg | SBP−DNPSBP−DNP |
| Relative dicrotic notch pressure | rDNP | mmHg | DNP−DBPDNP−DBP |
| Relative diastolic peak pressure | rDPP | mmHg | DPP−DBPDPP−DBP |
| Durations | | | |
| Relative duration systolic upstroke | rT_up | % | t(systolic peak)t(end)∗100%tsystolic peakt(end)∗100% |
| Relative duration systolic downstroke | rT_down | % | t(dicrotic notch)−t(systolic peak)t(end)∗100%tdicrotic notch−tsystolic peakt(end)∗100% |
| Relative duration diastole | rT_dia | % | t(end)−t(dicrotic notch)t(end)∗100%tend−tdicrotic notcht(end)∗100% |
| Heart rate | Hr | /min | 1(t(end)∗601(tend∗60 |
| Areas |  |  |  |
| Relative beat area | rA_beat | mmHg*s | ∫(p−DBP)(start:end)∫(p−DBP)(start:end) |
| Relative systolic area | rA_sys | mmHg*s | ∫(p−DBP)(start:systolic peak)∫(p−DBP)(start:systolic peak) |
| Relative diastolic area | rA_dia | mmHg*s | ∫(p−DBP)(dicrotic notch:end)∫(p−DBP)(dicrotic notch:end) |
| Relative area ratio | rA_ratio | - | rA_diarA_sysrA_diarA_sys |
| Slopes |  |  |  |
| Maximum slope | dP/dt max | mmHg/s | max (p.(start:systolic peak))max (p.(start:systolic peak)) |
| Systolic downstroke slope | S_down | mmHg/s | mean (p.(systolic peak:dicrotic notch))mean (p.(systolic peak:dicrotic notch)) |
| Diastolic runoff slope | S_dia | mmHg/s | mean (p.(dicrotic notch:end))mean (p.(dicrotic notch:end)) |

*p = pressure signal, t = time vector*

Table A2 - Change in waveform features per event

|  | **Fluid bolus (n=92)** | | **Norepinephrine (n=91)** | | **Dobutamine (n=35)** | |
| --- | --- | --- | --- | --- | --- | --- |
| **Feature** | Estimate change | p-value | Estimate change | p-value | Estimate change | p-value |
| **Pressures [mmHg]** | | | | | | |
| SBP | 4.5 [2.1-6.9] | **<0.001** | 4.6 [0.6-8.5] | 0.023 | 11.4 [0.2-22.6] | 0.046 |
| ANP | 4.3 [1.2-2.0] | **<0.001** | 4.2 [0.9-7.5] | 0.012 | 9.5 [-1.0-19.9] | 0.075 |
| DNP | 1.6 [0.1-3.1] | 0.034 | 3.1 [1.0-5.2] | **0.004** | 5.7 [-1.0-12.3] | 0.095 |
| DPP | 1.7 [0.3-3.1] | 0.021 | 3.3 [1.0-5.6] | **0.005** | 5.9 [-1.7-13.6] | 0.130 |
| DBP | 1.1 [0.1-2.0] | 0.029 | 1.8 [0.3-3.4] | 0.018 | 4.2 [-1.6-10.1] | 0.159 |
| MAP | 2.1 [0.8-3.4] | **0.002** | 2.7 [0.6-4.9] | 0.014 | 6.6 [-0.6-13.8] | 0.071 |
| **Relative pressures [mmHg]** | | | | | | |
| PP | 3.5 [1.7-5.2] | **<0.001** | 2.7 [0.1-5.4] | 0.045 | 7.2 [0.3-14.1] | 0.042 |
| AP | -0.2 [-1.3-0.9] | 0.748 | 0.3 [-1.0-1.6] | 0.677 | -3.0 [-4.5- -1.6] | **<0.001** |
| rANP | 3.3 [1.6-4.9] | **<0.001** | 2.3 [0.3-4.4] | 0.024 | 5.2 [-1.1-11.6] | 0.106 |
| DSP | 2.9 [1.4-4.4] | **<0.001** | 1.5 [-0.7-3.7] | 0.172 | 5.7 [0.1-11.3] | 0.045 |
| rDNP | 0.5 [-0.2-1.3] | 0.157 | 1.2 [0.5-2.0] | **0.001** | 1.4 [-0.7-3.5] | 0.177 |
| rDPP | 0.6 [-0.1-1.3] | 0.091 | 1.4 [0.5-2.4] | **0.003** | 1.7 [-1.6-5.1] | 0.316 |
| **Durations [%]** | | | | | | |
| rT_up | -0.4 [-0.7- -0.2] | **0.002** | -0.6 [-0.9- -0.2] | **0.004** | -0.2 [-1.6-1.3] | 0.823 |
| rT_down | 0.8 [0.2-1.3] | **0.006** | -0.6 [-1.2-0.0] | 0.062 | 0.5 [-1.8-2.8] | 0.664 |
| rT_dia | -0.3 [-0.9-0.3] | 0.282 | 1.1 [0.4-1.7] | **0.002** | -0.4 [-3.0-2.3] | 0.793 |
| Hr [/min] | -1.2 [-1.9- -0.4] | **0.002** | -0.6 [-1.6-0.3] | 0.182 | 4.1 [-0.2-8.5] | 0.062 |
| **Areas [mmHg*s]** | | | | | | |
| rA_beat | 1.1 [0.5-3.2] | **<0.001** | 0.9 [0.1-1.6] | 0.023 | 1.3 [-0.5-3.2] | 0.163 |
| rA_sys | 0.9 [0.5-1.3] | **<0.001** | 0.5 [-0.0-1.0] | 0.073 | 1.2 [-0.1-2.4] | 0.067 |
| rA_dia | 0.2 [-0.0-0.4] | 0.102 | 0.4 [0.1-0.6] | **0.003** | 0.2 [-0.7-1.0] | 0.699 |
| rA_ratio [ ] | -0.02 [-0.03- -0.01] | **0.004** | 0.04 [0.01-0.06] | **0.002** | 0.03 [-0.09-0.15] | 0.628 |
| **Slopes [mmHg/s]** | | | | | | |
| dP/dt max | 57.0 [25.5-88.6] | **<0.001** | 45.4 [3.0-87.8] | 0.036 | 166.2 [48.8-283.6] | **0.006** |
| S_down | -6.0 [-13.8-1.9] | 0.135 | -9.5 [-21.3-2.3] | 0.116 | -27.9 [-61.7-5.8] | 0.105 |
| S_dia | -0.7 [-1.9-0.5] | 0.272 | -1.8 [-3.1- -0.6] | **0.005** | -3.0 [-10.5-4.5] | 0.434 |

*Values presented as estimated change due to the hemodynamic treatment from a linear model with generalized estimating equations and a 95% confidence interval. P-values in bold are considered significant (p<0.01), features with blue shading are considered specific.*

Table A3 - Change in waveform features compared between events

|  | **Fluid bolus (n=92)** | **Norepinephrine (n=91)** | **Dobutamine (n=35)** |  |
| --- | --- | --- | --- | --- |
| **Feature** | Estimate change | Estimate change | Estimate change | p-value |
| **Pressures [mmHg]** | | | | |
| SBP | 4.5 [2.1-6.9] | 4.6 [0.6-8.5] | 11.4 [0.2-22.6] | 0.492 |
| ANP | 4.3 [1.2-2.0] | 4.2 [0.9-7.5] | 9.5 [-1.0-19.9] | 0.628 |
| DNP | 1.6 [0.1-3.1] | 3.1 [1.0-5.2] | 5.7 [-1.0-12.3] | 0.266 |
| DPP | 1.7 [0.3-3.1] | 3.3 [1.0-5.6] | 5.9 [-1.7-13.6] | 0.261 |
| DBP | 1.1 [0.1-2.0] | 1.8 [0.3-3.4] | 4.2 [-1.6-10.1] | 0.415 |
| MAP | 2.1 [0.8-3.4] | 2.7 [0.6-4.9] | 6.6 [-0.6-13.8] | 0.430 |
| **Relative pressures [mmHg]** | | | | |
| PP | 3.5 [1.7-5.2] | 2.7 [0.1-5.4] | 7.2 [0.3-14.1] | 0.489 |
| AP | -0.2 [-1.3-0.9] | 0.3 [-1.0-1.6] | -3.0 [-4.5- -1.6] | **0.001** |
| rANP | 3.3 [1.6-4.9] | 2.3 [0.3-4.4] | 5.2 [-1.1-11.6] | 0.594 |
| DSP | 2.9 [1.4-4.4] | 1.5 [-0.7-3.7] | 5.7 [0.1-11.3] | 0.284 |
| rDNP | 0.5 [-0.2-1.3] | 1.2 [0.5-2.0] | 1.4 [-0.7-3.5] | 0.260 |
| rDPP | 0.6 [-0.1-1.3] | 1.4 [0.5-2.4] | 1.7 [-1.6-5.1] | 0.261 |
| **Durations [%]** | | | | |
| rT_up | -0.4 [-0.7- -0.2] | -0.6 [-0.9- -0.2] | -0.2 [-1.6-1.3] | 0.877 |
| rT_down | 0.8 [0.2-1.3] | -0.6 [-1.2-0.0] | 0.5 [-1.8-2.8] | **0.002** |
| rT_dia | -0.3 [-0.9-0.3] | 1.1 [0.4-1.7] | -0.4 [-3.0-2.3] | **0.004** |
| Hr [/min] | -1.2 [-1.9- -0.4] | -0.6 [-1.6-0.3] | 4.1 [-0.2-8.5] | 0.049 |
| **Areas [mmHg*s]** | | | | |
| rA_beat | 1.1 [0.5-3.2] | 0.9 [0.1-1.6] | 1.3 [-0.5-3.2] | 0.849 |
| rA_sys | 0.9 [0.5-1.3] | 0.5 [-0.0-1.0] | 1.2 [-0.1-2.4] | 0.336 |
| rA_dia | 0.2 [-0.0-0.4] | 0.4 [0.1-0.6] | 0.2 [-0.7-1.0] | 0.293 |
| rA_ratio [ ] | -0.02 [-0.03- -0.01] | 0.04 [0.01-0.06] | 0.03 [-0.09-0.15] | **<0.001** |
| **Slopes [mmHg/s]** | | | | |
| dP/dt max | 57.0 [25.5-88.6] | 45.4 [3.0-87.8] | 166.2 [48.8-283.6] | 0.161 |
| S_down | -6.0 [-13.8-1.9] | -9.5 [-21.3-2.3] | -27.9 [-61.7-5.8] | 0.428 |
| S_dia | -0.7 [-1.9-0.5] | -1.8 [-3.1- -0.6] | -3.0 [-10.5-4.5] | 0.379 |

*Values presented as estimated change due to the hemodynamic treatment from a linear model with generalized estimating equations and a 95% confidence interval. P-values in bold are considered significant (p<0.01).*
